# Charge Transport in Conjugated and Saturated Hydrocarbons: Comparing Ballistic and Cotunneling Contributions

**DOI:** 10.1021/acs.jpca.3c05869

**Published:** 2023-12-15

**Authors:** Hugo Cabrera-Tinoco, Augusto C. L. Moreira, Luis Borja-Castro, Renato Valencia-Bedregal, Crispin H. W. Barnes, Luis de los Santos Valladares

**Affiliations:** † Facultad de Ingeniería, Universidad Continental, Lima 15311, Perú; ‡ Núcleo Interdisciplinar em Ciências Exatas e da Natureza (NICEN), Universidade Federal de Pernambuco, 55014-900 Caruaru − PE, Brazil; § Laboratorio de Cerámicos y Nanomateriales, Facultad de Ciencias Físicas, Universidad Nacional Mayor de San Marcos, Ap. Postal 14-0149 Lima, Perú; ∥ Cavendish Laboratory, Department of Physics, University of Cambridge, J. J Thomson Avenue, Cambridge CB3 0HE, U.K.

## Abstract

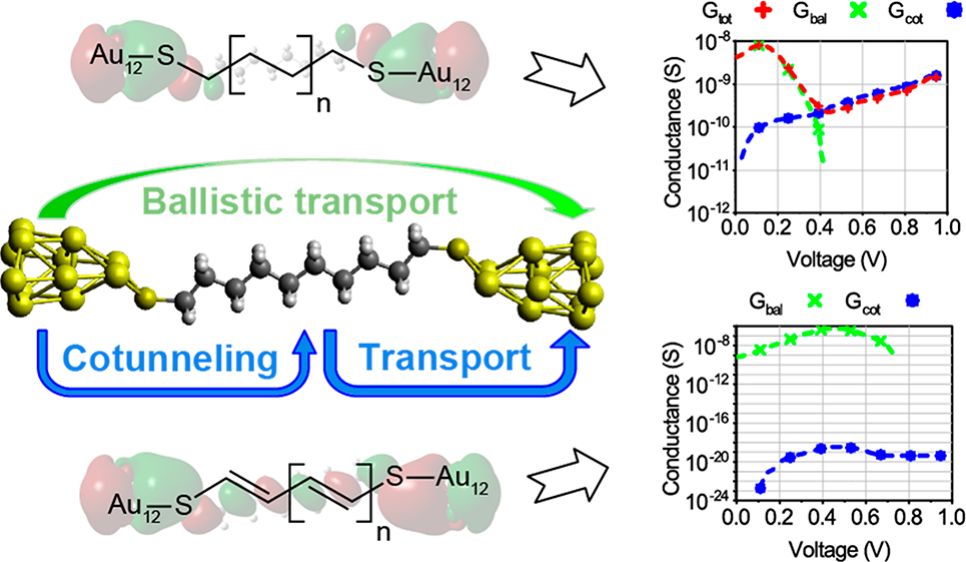

The comparison between electrical transport in C_*n*_H_2*n*+2_S_2_ alkane and C_*n*_H_*n*+2_S_2_ alkene (*n* = 4, 6, 8, 10) is studied by using a generalized Breit-Wigner approach and considering coherent transport mechanisms and eventual changes in the state of charge (i.e., cotunneling processes) for both molecules. In general, the conductance of alkanes tends to be smaller than that of similar-sized alkenes. However, cotunneling processes have an important participation in the overall transport in the case of alkanes but not for the alkene family. The progressive changes in both the eigenenergies of the relevant frontier molecular orbitals of the charged species and their spatial localization play decisive roles in the observed differences. While the molecular orbitals of the charged species of the conjugated molecules are hardly affected by the applied voltage, their saturated counterparts are quite sensitive to the external field. With this, successive avoided-crossing events between the molecular orbitals of the single-charged alkane molecules can lead to the appearance of nonballistic conduction channels that make no negligible contributions to the molecular transport.

## I Introduction

A hallmark of digital-age technology, the progressive miniaturization of electronic components, has been a major driving force for the development of condensed matter physics at the nanoscale. In this regard, the exploration of the physical and chemical properties of organic compounds, with their inherent low dimensionality, appears as a viable alternative for the further integration of electronic components into functional molecular devices, a prospect foreshadowed by the present complexity of biological mechanisms.^1−5^ Nowadays, the most used tool for the study of the electronic transport through an organic molecule connected to two metallic electrodes is the combination of the nonequilibrium Green function (NEGF) formalism with density functional theory (DFT) procedures,^6^ which has been used to describe the charge transport in a wide variety of molecular systems.^7−10^ However, in its usual form, this technique describes the problem of transport in the representation of single-electron states, which is inadequate to explain phenomena where electron correlation plays an important role.^11^

There are many ways of introducing many-body correlations effects, such as the DFT + Σ approximation that in an *ad hoc* manner corrects the self-energy error in the DFT calculations^12,13^ and the combination between NEGF and the GW approximation.^11,12^ However, the computational costs associated with both types of treatment impede the description of more realistic systems of interest.^14^ In lower dimensions, one must properly account for quantum effects in which the interaction between electrons dominates the transport. Such are the cases of Coulomb blockades^15,16^ and quantum interference^17,18^ and oxidation/reduction effects,^19−21^ examples of phenomena that cannot be properly handled by NEGF-DFT methods. Recently, while examining an external electric effect on organometallic molecules, Schwarz identified a hysteretic behavior in the current–voltage curves and the manifestation of switch-type characteristics.^22^ He attributed this switching effect to the participation of localized molecular orbitals in the transport process, through oxidation/reduction mechanisms^22^ responsible for the observed nonlinear characteristics.^23^

To treat this problem, Migliore and Nitzan^24,25^ used a model based on the Marcus theory^26^ and NEGF-DFT methods and described the transport as being composed of both a fast mechanism, which dominates the driving, and a slow one that allows changes in the charge state of the molecule during transport. This model addresses the oxidation and reduction processes in terms of electron-transfer reaction rates rather than using the language of Green functions. However, while avoiding the drawbacks of an ab initio approach, this procedure obscures the relationship between the fast (coherent tunneling) and slow (incoherent hopping) mechanisms. Also, in calculating the *I*–*V* curves, the parameters of the Marcus theory were assumed to be invariant, no matter the strength of the field applied.^27−29^

At the same time, other formalisms that describe hopping-based transport, such as the rate equation^30^ and quantum master equation approaches,^31−33^ are restricted to the regime in which the coupling of the molecule with the electrodes is weak, which makes them inadequate to describe the processes of coherent tunneling or ballistic transport. Hence, novel models could adequately describe processes in which ballistic and hopping-type mechanisms simultaneously contribute to the overall charge transport.

In a previous work, we have proposed a formalism that extends the scope of the Breit-Wigner treatment of the transmission function by including the participation of the neutral, cationic, and anionic species during the transport.^34^ In that treatment, the molecule can maintain its charged state (i.e., when one has “ballistic” transport) or receive or lose one electron (through coupling processes that involve more than one state of charge).

For each of the three possible charge states, we used DFT to describe the electronic structure of the extended molecule (i.e., the complex formed by the organic molecule plus the two terminal metallic clusters to where it is attached)^35^ for different voltage values. In this manner, we were able to determine the spatial localization of the relevant molecular orbitals and use the corresponding eigenvalues as the necessary parameters for the implementation of the formalism. It was then possible to follow the change of the electronic structure of the three species with increasing voltage and achieve a more detailed understanding of the microscopic processes involved in the charge transport. In this aspect, it is especially relevant to note the occurrence of avoided-crossing situations between neighboring molecular orbitals, at specific values of the applied field, since they could play a crucial role in the cotunneling contributions to the overall transport.^36^

Several reports dedicated to the investigation of the charge transport in saturated^37−40^ and conjugated hydrocarbons^41−43^ provided the evidence that characteristic π-electron delocalization of the latter responds for their much higher conductivity.^44,45^ Obvious differences in the electronic structure exist even for similarly sized alkanes and alkenes. One could expect to be able to relate the distinct spatial localization of the frontier molecular orbitals of saturated and conjugated hydrocarbons to differences in the relative contributions of ballistic and cotunneling mechanisms to the overall charge transport in these molecules.

In the present paper, we use the generalized Breit-Wigner treatment^34^ to compare the charge transport across the C_*n*_H_2*n*+2_S_2_ alkanes and C_*n*_H_*n*+2_S_2_ alkenes, with (*n* = 4, 6, 8, 10). In Section II, we briefly review the formalism used to calculate the current and conductance of the different molecules examined. In Section III, we show the corresponding current and conductance curves and then analyze the contributions of the ballistic and coupling mechanisms by detailing the cases of the decanedithiol and deca-1,3,5,7,9-pentaene-1,10-dithiol molecules. We present our conclusions in Section IV.

## II Methodology

We considered the organic molecule of interest as connected to two terminal electrodes. Then, Landauer’s formula^46−48^
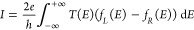
1where *f*_*L*_ and *f*_*R*_ are the Fermi distribution functions of the left and right electrodes, respectively, and *T*(*E*) is the transmission function, can be used to calculate the current traversing the system. As usual, *f*_*R,L*_ = *f*(*E* – *μ*_*R,L*_), where *μ*_*R*_ and *μ*_*L*_ are the chemical potentials of the electrodes, which define the Fermi window of the problem (i.e., only those molecular orbitals whose eigenvalues are within this range may participate in the charge transport). We have previously extended the Breit-Wigner approach and obtained a generalized expression for the transmission function that includes both ballistic and cotunneling contributions to the overall charge transport. According to our model, the transmission can be written as *T*(*E*) = ∑*T*_*n*_^*bal*^(*E*) + *T*_*n*_^cot^(*E*), where for the orbital with the eigenvalue *ζ*_*n*_ the contributions of the ballistic and cotunneling processes can be described by
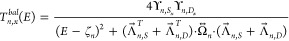
2and
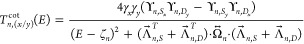
3respectively. In these equations, the 3 × 3 Ω⃡*n* matrix contains the information concerning the molecular orbitals that can participate in the transport, according to their occupancy and the nature of the charged species considered (with the subindexes 1, 2, and 3 being related to the cation, neutral, and anion species, respectively). In this manner, the Ω_11_, Ω_22_, and Ω_33_ elements are equal to 1, since they refer to processes in which the occupancy remains the same. For instance, Ω_22_ corresponds to the case where only the neutral molecular orbitals participate in the charge transport, and if the corresponding conduction channel involves an occupied molecular orbital, it will not receive or lose electrons during the charge transfer process. On the other hand, Ω_12_ and Ω_23_ can be 1 or 0, depending on the occupancy of the orbitals involved. For example, if the participating orbital of the neutral species is HOMO and the orbital of the anion species is also occupied, then the Ω_23_ element will be zero since both orbitals cannot have the same occupancy; otherwise, Ω_23_ is 1. The elements Ω_13_ and Ω_31_ must always be equal to zero, since they would correspond to processes in which charge would be transferred between the anionic and cationic species, and the participation of two electrons is not contemplated in our model. (See ref (34) for more details.)

The vector
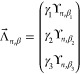
4expresses the coupling strength of the molecule with the β electrode, where once again the subscripts *x* = 1, 2, and 3 indicate the cationic, neutral, and anion charge state of the molecule, respectively, and ϒ_*n,βx*_ is the thermo-electronic coupling of the molecule with the electrode. Considering the probability of participation of each state of charge as fixed and given by γ_{*`x*=1,2,3}_^2^ so that γ_1_^2^ + γ_2_^2^ + γ_3_^2^ = 1, we will assume a Poisson distribution so that γ_*x*(*k*)_^2^ = λ^*k*^*e*^*–λ*^/*k*!, where *k* is the number of charge carriers trapped in the molecule, and λ = ∑_*i*=1_^*N*^(*R*_*i*_/*R*_*M*_ – 1), where *R_M_* [*R_i_*] are the lengths of the bonds between consecutive carbon atoms of the benzene molecule [molecule to be studied]. One can understand λ as a measure of the degree of aromaticity^49^ of the molecule to be studied and consider that *k* = 0 for the neutral subspace and *k* = 1 for the anion and cation subspaces, with the normalized probabilities
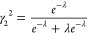
5and
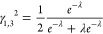
6The thermo-electronic coupling term ϒ_*n,βx*_ is defined as the product between the electronic coupling molecule–electrode (Γ_*n,β*_*x*__ = *i*[Σ_*n,β*_*x*__ – Σ_*n,β*_*x*__^†^]) and the thermal expansion function *F*_*T*_.^46^

In the actual calculation, we used the extended molecule approach^35^ to model the modifications of the electronic structure of the molecule of interest, which was terminally coupled to two metallic clusters representing the electrodes. We initially optimized the geometry of the extended molecule, by assuming the spatial arrangement of the metal atoms in each cluster to be fixed and follow the so-called French hat geometry,^50^ and then determined the optimal length of the extended molecule after relaxing the atomic configuration of the organic part. (See the Supporting Information for more details.) We then used the DFT method to calculate the electronic structure of the extended molecule when it was subjected to different values of an externally applied electric field. We adopted the hybrid functional B3LYP included in the Gaussian 03 program,^51^ with the 6-31G (d, p) [LANL2DZ] basis set being used for the organic part [metallic clusters]. Since the Gaussian 03 program allows only a minimum incremental step of 10^–4^ a.u. for the electric field, we linearly interpolated intermediate points of the autoenergies and coupling parameters (defined in eq 7) of the molecular orbitals as necessary to generate a finer grid. (In the curves of current and conductance, the noninterpolated points will be highlighted.)

**Figure 1:**
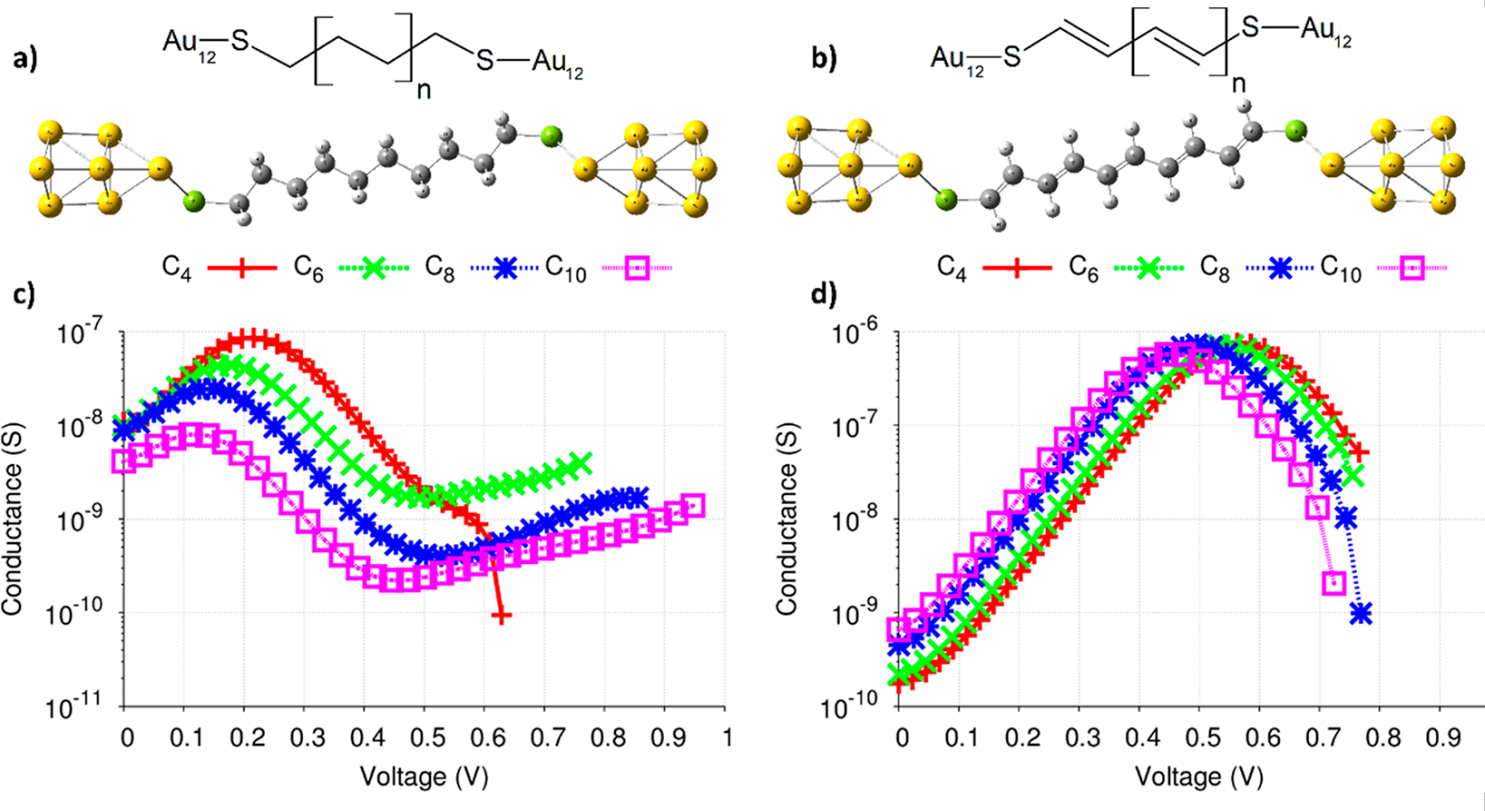
Structures of the (a) decanedithiol and (b) deca-1,3,5,7,9-pentaene-1,10-dithiol molecules attached to a metallic cluster at each extreme (extended molecule). Variation of the conductance for molecules of the (c) alkane and (d) alkene families as a function of the applied voltage.

In Figure 1, we schematically present the optimized structures of the decanedithiol (Figure 1a) and deca-1,3,5,7,9-pentaene-1,10-dithiol (Figure 1b) molecules along with their corresponding chemical structures.

Once the quantum chemistry calculation was completed, one could quantify the spatial distribution of the molecular orbitals of the extended molecule through the coupling parameter
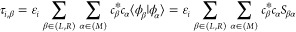
7where *ε*_*i*_ and *c*_*β,α*_ are the corresponding eigenvalues and projections of the molecular orbital on the atomic orbital basis {|*ϕ*_*β,α*_⟩}, respectively. This coupling parameter can be understood as an estimate of the degree of sharing of the spatial distribution between the molecule and the metallic clusters of the *i*th molecular orbital. Once this information is extracted from the quantum chemistry calculations, we can apply the generalized Breit-Wigner formula (eqs 2 and 3) to calculate the transmission function of the ballistic and cotunneling components of the total current.

In addition, an important parameter to analyze is the degree of resonance
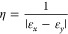
8which is a measure of the closeness of the eigenvalues ε_*x*_ and ε_*y*_ of the orbitals of two different charged species.

## III Results and Discussion

Before discussing the results obtained using the formalism described in the previous section, the comparison of the GBW formalism with the Anderson model (AM) has been evaluated. The AM is a standard and well-established formalism that, applied to an impurity or a quantum dot with a single level, describes the Coulomb charge effects of a single electronic state coupled to two electrodes that can be in three charge states during electronic transport.^52,53^ The Hamiltonian that defines the AM is given as *Ĥ* = *Ĥ*_*A*_ + *Ĥ*_*R*_ + *Ĥ*_*L*_ + *Ĥ*_*A,R*_ + *Ĥ*_*A,L*_. The Hamiltonian for the impurity is *Ĥ*_*A*_ = ∑_σ_ε_σ_c_σ_^†^*c*_σ_ + *Un*_↑_*n*_↓_, where *ε*_*σ*_,U, *n*_↑_, and *n*_↓_ are the single-level energy, the intrasite Coulomb repulsion energy, and the number operators for the two possible spin orientations, respectively. *Ĥ*_*R*_ and *Ĥ*_*L*_ are the Hamiltonians of the electrodes (right and left). *Ĥ*_*A*,*R*_ and *Ĥ*_*A,L*_ describe the charge transfer between the impurity and both electrodes. In summary, the AM Hamiltonian has a Fock space of four eigenstates according to the occupation of the single level with the corresponding eigenenergies: *E*_1_ = 0, *E*_2_ = *E*_3_ = ε_0_, and *E*_1_ = 2ε_0_ + *U* (see Figure S1(a)). The details of those discussed above can be found in the literature.^47,52,53^ The *I*–*V* curves generated by the AM consist of three characteristic plateaus for each charge state, and each jump is located at 0, 2ε_0_, and 2(ε_0_ + *U*) in the voltage domain.

We then proceeded to compare the results obtained with those of our generalized Briet-Wigner approach. In this sense, a single energy level system that can be in three charge states—cation (N – 1), neutral (N), and anion (N + 1)—was adopted. Each state of charge has a different eigenenergy value as shown in Figure S1(b). The parameters used for the two approaches are Γ_*R*_ = Γ_*L*_ = 1.5 meV, ε_0_ = 70 meV, and *U* = 120 meV. It can be seen in Figure S1(d,c) that the values of the probabilities γ that reproduce the profile of the *I*–*V* curves generated by the AM are γ_*N*–1_^2^ = 0, γ_*N*_^2^ = 0.55, and γ_*N*+1_^2^ = 0.45 for 40 and 130 K. It should be noted that a similar analysis was carried out in ref (34) with different values for the parameters and temperature with concordant results. However, the same set of probabilitie*s* γ continues to reproduce the AM results. This allows us to affirm that the GBW approach describes the main characteristics of the AM results even with its differences.

We calculated how the current traversing the alkane and alkene molecules considered (Figure S2) and the corresponding conductance *G* = d*I*/d*V* (Figure 1c) would change as the applied voltage is progressively increased. The conductance curves were adjusted to obtain the parameter β for alkane and alkene molecules according to the relationship *G* = *G*_0_ exp(−*βL*), where β and *G*_0_ are experimentally determined parameters and *L* is the molecular size. This analysis is shown in section SI-2. In Figure 1c, one can see that for most of the alkane molecules (the special case of the C4 alkane will be discussed later), the conductance initially increases and reaches a first maximum, experiencing then a second increase for voltages greater than ∼0.5 V. We will show how this behavior can be explained in terms of spatial delocalization of the molecular orbitals involved in the transport, with the maximum conductance values being observed when the MOs involved are mostly delocalized.

Unlike alkanes, alkenes are conjugated molecules, and their π orbitals are spatially delocalized. Hence, one could expect them to exhibit a higher level of conductance than alkane molecules of similar size. In Figure 1d, we show that the calculated conductance for the alkene molecules considered can reach values in the 600 to 750 nS range, which are almost 1 order of magnitude above those found for the corresponding members of the alkane family. For example, while a maximum conductance of 85.93 nS was calculated for the C4 alkane, in the case of the C4 alkene, this maximum is estimated to be 756.48 nS. As a general trend, our results confirm that after a certain threshold voltage, the alkenes should indeed be better conductors than the alkane molecules. Even so, one can also observe that in a limited voltage range, the conductance for the alkanes can become higher than that of similar alkenes. For instance, for *V* < 0.31 V the conductance of the C4 alkane is greater than that of the C4 alkene. As we will discuss soon, this fact can be understood in terms of the extent of spatial localization of the corresponding frontier orbitals. A comparison between the corresponding conductance curves is shown in Figure S3.

It is important to note that the experimental evidence to date indicates that conjugated molecules have higher conductance than saturated molecules of similar sizes over any voltage range.^45^ In this sense, our results do not seem to be consistent with it. However, we have not found in the literature a direct comparison between alkanes and linear alkenes, as we have wanted to do in this article. One of the possible causes of our results is the chemical model. However, we performed the calculations with PBE,^54^ and the results were similar to the B3LYP ones. Therefore, we rule out that possibility.

On the other hand, it has been shown that the electrical response of molecular systems has a very important dependence on the anchoring group and the coordination with the electrodes.^55^ This can cause variations of several orders of magnitude.^56^ As mentioned in the previous section, our molecular junction model takes 2 clusters of 12 atoms to model the interaction between the molecule and the electrodes. In this sense, the literature shows that the electronic structure of metallic clusters is related to the number of atoms, and, for example, there may be variations in the HOMO–LUMO gap of more than 1 eV for clusters a few atoms apart.^57^ Because of this, we presume that our results are due to the cluster model that we used in our calculations. Although our results at low voltages do not fit the experimental evidence, it is worth noting that this changes with increasing voltage, as mentioned at the beginning of this section. In addition, as will be seen later, the model describes the spacial localization wave function of the frontier orbitals in a good way.

**Figure 2:**
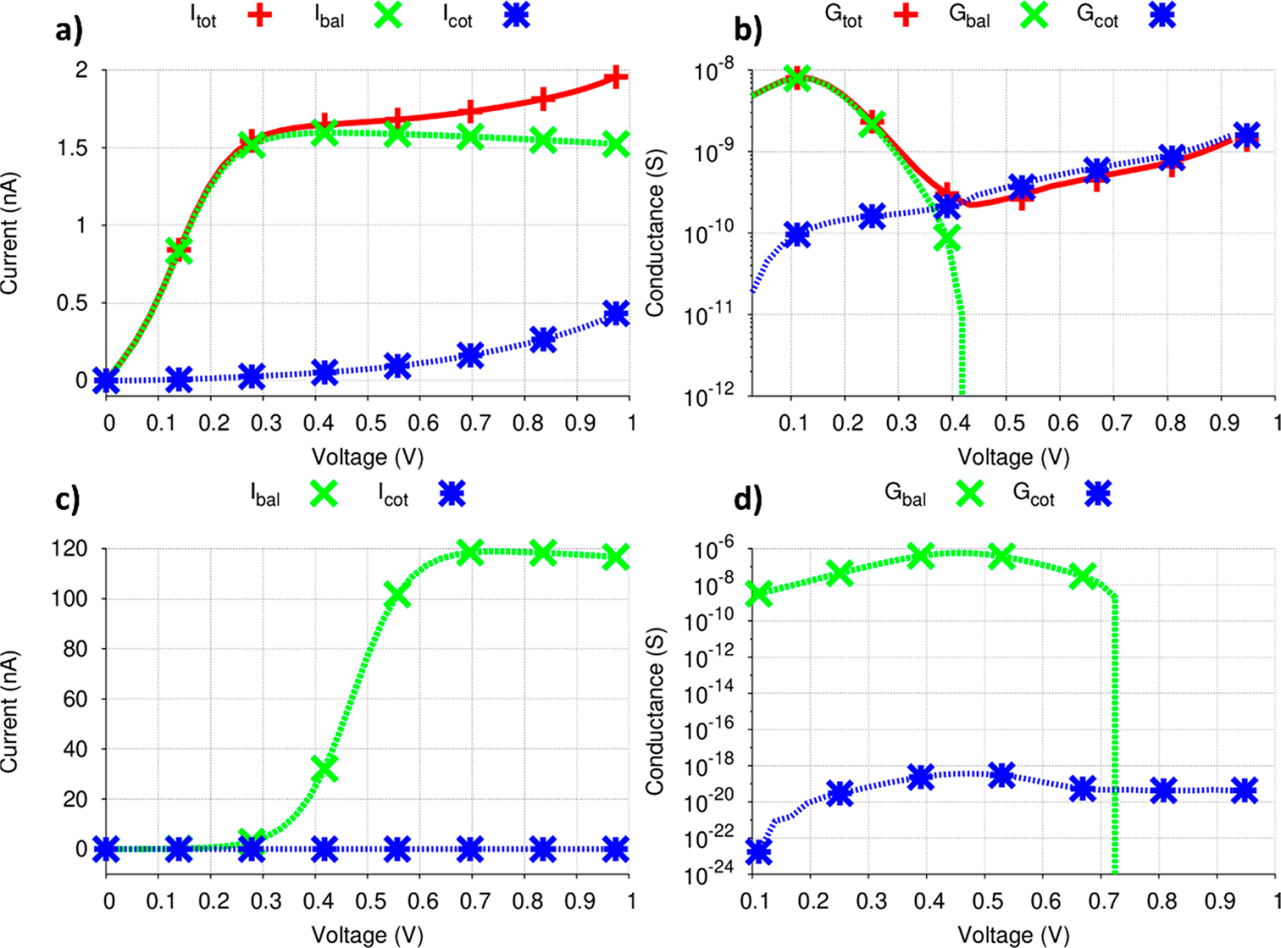
(a) Total, ballistic, and coupling current and (b) conductance of the decanedithiol (C10 alkane) molecule. (c) Ballistic and cotunneling current and (d) conductance of the 1,3,5,7,9-decepenteneddithiol (C10 alkene) molecule.

Our treatment allows the calculation of the individual ballistic and cotunneling currents by the use of eqs 2 and 3, respectively. We have then analyzed the characteristics of these two mechanisms and compared their relative contributions to the overall transport. Here, we discuss the results for the C10 alkane and C10 alkene molecules (Figures 2 and 4).

Let us consider the C10 alkane case. For this molecule, one can observe in Figure 2(a,b) that while at low bias the ballistic contribution is dominant, for *V* > 0.4 V the ballistic current begins to decrease, with the cotunneling contribution increasing noticeably. The corresponding ballistic process involves the participation only of the neutral subspace, with the HOMO and LUMO playing the role of conduction channels; we have confirmed this by observing the ballistic transmission function (see Figure S4(a)). These orbitals can participate in the transport because their eigenvalues lie in the chosen Fermi window and their wave function is spatially delocalized over the extended molecular structure, characteristics that are still preserved^34^ after the external field is switched on. All other orbitals with energies in the Fermi window have wave functions that are localized in some regions of the extended molecule, thus precluding their participation in ballistic conduction.

We can also analyze the behavior of the ballistic current in terms of coupling parameters *τ*_*L*_ and *τ*_*R*_. In the presence of an external electric field, while remaining delocalized the neutral HOMO and LUMO wave functions couple differently to the two electrodes. For instance, while at zero bias τ_*L*_^*HOMO*^ = τ_*R*_^*HOMO*^ = 2.55 × 10^–3^, at *V* = 0.14 V we have τ_*L*_^*HOMO*^ = 2.49 × 10^–3^ and τ_*R*_^*HOMO*^ = 2.60 × 10^–3^ so that a slight localization of the wave function on the right side of the extended molecule occurs as the intensity of the applied field progressive increases. A similar effect is observed for the LUMO, but with increasing localization occurring on the left side of the extended molecule. The ballistic current reveals the occurrence of a situation of negative differential resistance (NDR) since after reaching a maximum of 1.59 nA at 0.44 V the current decreases to 1.52 nA at 0.98 V (Figure 2(a)). Therefore, the corresponding ballistic conductance (Figure 2(b)) exhibits a maximum at 0.11 V and then decreases. At larger voltages, no conduction channels other than the neutral HOMO and LUMO contribute to the ballistic transport (since only those two molecular orbitals have energies inside the Fermi window). This causes a decrease in the conductance, which finally assumes negative values (not seen in Figure 2(b) due to the semilogarithmic scale adopted) at voltages greater than 0.44 V. Although different models have been used in the literature to explain the occurrence of NDR in molecular systems,^58^ the analysis of this effect in terms of the role played by the frontier molecular orbitals remains little explored.

As we have indicated before, a feature of our treatment is to provide for a possible contribution of nonballistic processes to the overall transport. Although orbitals with a spatially localized wave function cannot participate in ballistic processes, they could be involved in cotunneling transport. In these latter processes, the charge transfer between the electrodes consists of a more complex conduction channel that involves the simultaneous participation of orbitals of differently charged species of the molecule considered. Namely, in our case, the cotunneling processes we are interested in are those in which a transient anion [cation] species is formed after the electron [hole] is transferred from the electrode to the LUMO [HOMO] of the neutral species.

We have analyzed the cotunneling transmission function (as calculated according to eq 3) in search of identifying the molecular orbitals of the occupied anion [unoccupied cation] that could form a cotunneling conduction channel with the LUMO [HOMO] of the neutral species (see Figure S4). For this, we examined the degree of resonance (eq 8) η of the two MOs involved and the coupling parameters (eq 7) *τ*_*L*_ and *τ*_*R*_ of each of these orbitals on the right and left sides of the extended molecule. We have found that anion MOs that are involved in the transport (as identified by the form of the cotunneling transmission function, see Figure S5) have their wave function mostly localized on the left and central parts of the extended molecule so that the transport will occur with the assistance of the neutral LUMO (whose wave function is delocalized over the entire system). As for the relevant cation MOs, whose wave functions are localized on the right and center of the extended molecule, the corresponding conduction channels involve the participation of the delocalized neutral HOMO.

As one can observe in Figure 2(a), the rate of increase of the cotunneling current is initially lower than that of the ballistic one, a situation that is reversed at higher voltages. The importance of the coupling contribution can be better appreciated by examining the profile of the corresponding conductance curve (Figure 2(b)). While the ballistic contribution dominates the conductance behavior at voltages smaller than 0.36 V, it progressively decreases and reaches negative values for *V* ≥ 0.44 V. At higher voltages, the cotunneling processes dominate the overall transport.

For the alkane molecules considered (except for the C4, see later), a qualitatively similar behavior was observed (i.e., while the transport is dominated by ballistic processes at low voltages, the cotunneling contribution becomes progressively more important as the intensity of the applied field is increased). As for the C4 alkane, in this case, the degree of resonance between the anion and neutral MOs begins to decrease for *V* ≥ 0.4 V, causing the marked decrease of the conductance seen in Figure 1c.

We performed a similar investigation for molecules of the alkene family. In Figure 2(c,d), where we present the results for the C10 alkene, one can observe that the transport is entirely dominated by ballistic processes (Figure 2(c)). For *V* ≥ 0.3 V, the ballistic current presents a pronounced increase while the coupling current is essentially zero. As one can observe in Figure 2(d), the cotunneling conductance is negligible relative to the ballistic contribution. For the entire voltage range examined, we have found that, contrary to what was observed for the alkane family, the relevant alkene C10 anion and cation MOs (i.e., those that present a coupling parameter permitting a significant cotunneling contribution) have a very low degree of resonance with the neutral MOs. The ballistic conductance assumes negative values for *V* ≥ 0.72 V, and this is reflected in the abrupt drop of the corresponding semilogarithmic curve in Figure 2d.

The same pattern is also present in all of the other alkene molecules investigated.

**Figure 3:**
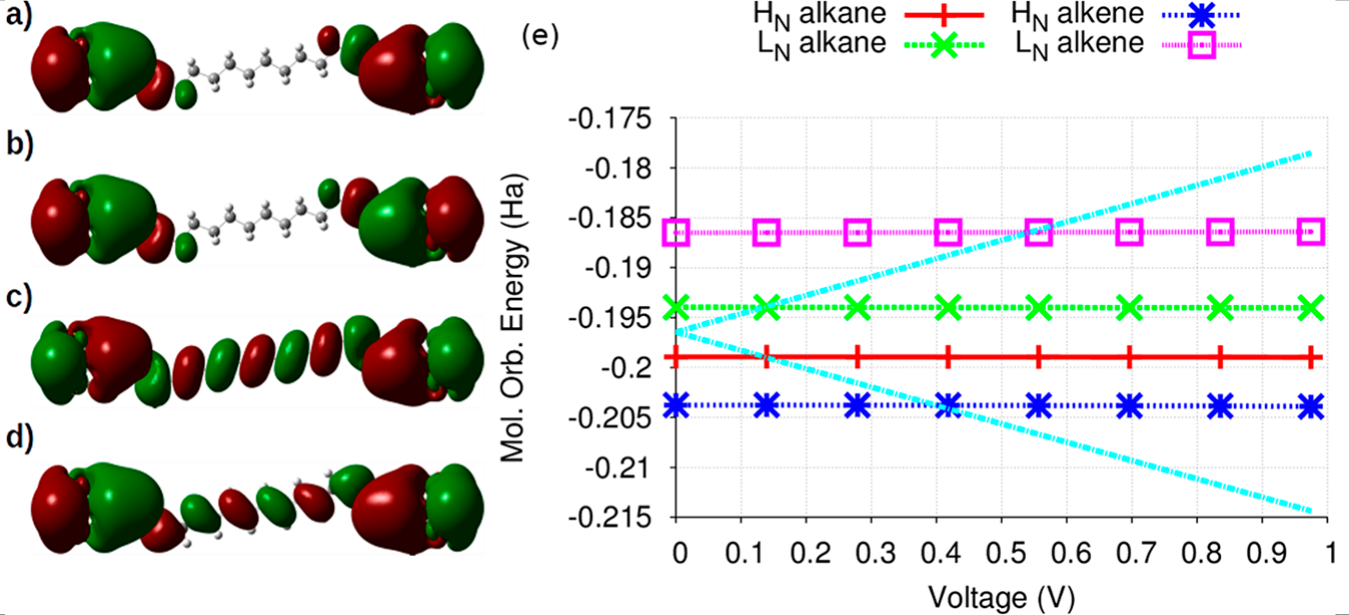
Map of the spatial localization (isovalue = 0.005) of the neutral C10 alkane (a) HOMO and (b) LUMO and the neutral C10 alkene (c) HOMO and (d) LUMO. (e) Variation of the eigenvalues of these neutral frontier orbitals as a function of voltage. The light-blue dashed lines indicate the progressive enlargement of the Fermi window.

Proceeding further in the investigation of the differences in the nature of the charge transport of alkanes and alkenes, we examined the progressive changes in the electronic structure of the C10 molecules of the two families observed with an increase in the external voltage. As mentioned before, in each case the ballistic contribution occurs through the HOMO and LUMO orbitals of the corresponding neutral species (*H*_*N*_ and *L*_*N*_). In Figure 3, we show the spatial localization of these two frontier MOs, where one can observe the more delocalized character of alkenes *H*_*N*_ and *L*_*N*_. Also, the HOMO–LUMO gap of these two molecules remains essentially constant in spite of changes in the field intensity (Figure 3(e)), with a much smaller gap observed for the C10 alkane. As a consequence, the energies of the frontier orbitals of the alkane molecule start to lie inside the Fermi window at a much lower bias value than in the case of the C10 alkene (namely, while at *V* ≈ 0.3 V the alkane *H*_*N*_ and *L*_*N*_ are totally inside the Fermi window, a situation that occurs only at *V* ≈ 0.7 V for the alkene *H*_*N*_ and *L*_*N*_). The behavior of the conductance for molecules of both families can be observed in Figure S3. This fact explains why higher values of the current and conductance are observed at lower voltages in the case of the alkane molecule. We have found an equivalent pattern when comparing other pairs of similarly sized molecules of the two families. As discussed above, this underestimation of the HOMO–LUMO gap in the case of alkanes is most likely due to the cluster model used in this work. Calculations performed with PBE show results similar to those shown above. With this, we rule out that these results are due to the functional effect.

**Figure 4:**
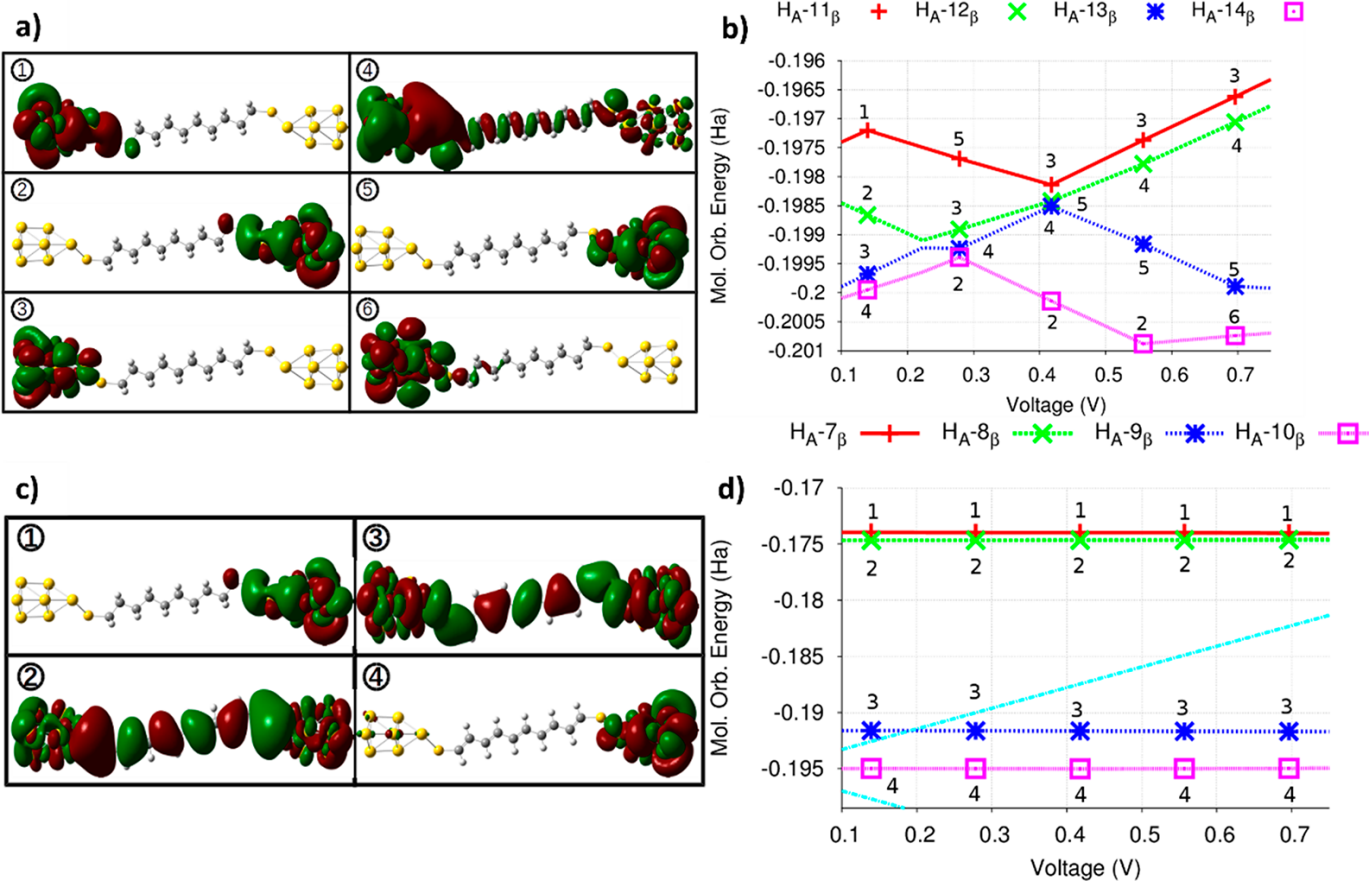
(a) Types of spatial localization (isovalue = 0.005) related to anion beta MOs in the Fermi window. (b) Evolution of the eigenenergies of these orbitals vs applied voltage. The numbers indicate how the type of localization changes with the voltage. (c) Types of spatial localization (isovalue = 0.005) of the deca-1,3,5,7,9-pentaene-1,4-dithiol (C10 alkene) anion beta MOs that lie in the Fermi window. (d) Eigenenergies of these orbitals vs applied voltage. The numbers indicate the type of localization of each orbital. The Fermi window is highlighted by dotted lines.

A less straightforward analysis is required for exploring the reasons for the distinct behavior of the cotunneling contribution in alkanes and alkenes. For instance, when the field is switched on, the beta MOs of the cation and anion C10 alkane (which are those whose energies lie in the Fermi window) become localized in different regions of the extended molecule (Figure 4). While six distinct types of spatial localization can be identified in the 0 < *V* < 1.0 V range, we will show that only one of them (type 4) appears suitable for allowing the opening of a channel of cotunneling conduction. This occurs because even though the corresponding wave function is spatially distributed only in the left metallic cluster and the organic part, in the cotunneling process, the neutral LUMO provides the necessary delocalization on the right side of the extended molecule.

As the external voltage is increased, the eigenvalues of the different molecular orbitals are modified, and at some specific bias, the eigenvalues of two MOs may approach each other, allowing the occurrence of an avoided-crossing situation that is accompanied by an interchange of their spatial localization.^34,59^ For instance, this is the case for *H*_*A*_ – 12_β_ and *H*_*A*_ – 13_β_ at 0.22 V (Figure 4 (b)). For *V* < 0.22 V, *H*_*A*_ – 12_β_ and *H*_*A*_ – 13_β_ have localizations of types 1 and 2, respectively, but after the avoided-crossing event, they reverse their spatial localization. In Figure 4(b), we also present the sequence of observed avoided-crossing instances. The localization of type 4 follows the sequence *H*_*A*_ – 14_β_ (0.14 V) → *H*_*A*_ – 13_β_ (0.27 V) → *H*_*A*_ – 12_β_ (0.55 V). This causes the MO eigenvalue with the localization of type 4 to progressively approach the eigenvalue of *L*_*N*_ (for instance, η is 167.5 and 628.93 at 0.14 and 0.97 V, respectively), thereby favoring the establishment of a cotunneling channel. Hence, as the energies of the orbitals participating in the cotunneling processes progressively change with the increasing voltage (Figure S6), an effective conducting channel (Ch) is formed (purple squares in Figure S6).

This rather complex chain of events lies behind the increasing relative contribution of the coupling mechanism to the overall transport as the voltage changes. A similar situation is identified for the anion alpha MOs of the C10 alkene, but in this case, the corresponding contribution to the transport is smaller due to the lower degree of resonance between the MOs of the neutral and charged species. Finally, we have found that a smaller number of avoided crossing situations exist for the cation MOs, with the spatial localization of the corresponding MOs being such that they make a smaller contribution to the cotunneling transport.

As for the other alkane molecules investigated, we have found that the C8 molecule exhibits characteristics similar to those identified in C10, with the anion MOs presenting the largest contribution to the cotunneling processes. As for C6, the anion and cation MOs give equivalent contributions. Finally, in the case of C4, the cation MOs give the largest contribution (0.87 nA at 0.68 V), while that of the anion orbitals saturates at a current value of 0.27 nA at *V* = 0.4 V. At this voltage, the degree of resonance between the neutral and anion MOs reaches its maximum value, a fact that explains the decrease in the conductance observed in Figure 1c.

We analyzed the electronic structure of the molecules of the alkene family in search of understanding why, for these conjugated systems, the cotunneling contributions are so much smaller than that found for the saturated alkanes. In Figure 4(c,d), we show both the different types of spatial localization of the relevant C10 alkene MOs and how their eigenenergies change as the voltage is progressively increased. We have found that although localization types 2 and 3, which are respectively related to *H*_*A*_ – 8_β_ and *H*_*A*_ – 9_β_, are suitable for contributing to cotunneling processes, these MOs exhibit a low degree of resonance with the neutral LUMO. At *V* = 0.13 *V*, the degrees of resonance of the *H*_*A*_ – 8_β_ and *H*_*A*_ – 9_β_ orbitals are 84.6 and 195.31 (a.u.)^−1^, respectively, and these values remain almost unchanged even at higher voltages (84.6 and 186.26 (a.u.)^−1^, respectively, at 0.97 V, which is the highest voltage value used for this molecule), different from what was observed in the case of alkane MOs (Figure S6). Then, the contribution of a pair of molecular orbitals to tunneling transport is correlated to the corresponding degree of their resonance. For molecules of the alkene family, this resonance remains small for the entire voltage range that we investigated. Besides, for a cotunneling conduction channel to be established between molecular orbitals of the charged and the neutral species, the corresponding eigenvalues must approach each other and their spatial localization must be complementary.

An important point to stress is that the most relevant difference we have found for the changes in the electronic structure of alkanes and alkenes induced by the progressive increase in the field intensity seems to be associated with the fact that avoided-crossing effects are absent for the MOs of the charged species of the latter type of molecules. As we have shown, in the case of alkanes, the occurrence of successive avoid-crossing events results in an increase in the degree of resonance between neutral and charged MOs, thus allowing no negligible participation of cotunneling mechanisms. As avoided-crossing events between neighboring MOs (with the consequent appropriate changes in the spatial localization) do not occur in the alkene molecules, for them, the charge transport will be dominated by ballistic processes. In addition, we have found that the molecular orbitals of the cation species have behavior similar to that of the corresponding anionic molecules, in which even MOs with adequate localization are not involved in cotunneling processes due to an insufficient degree of resonance with the neutral HOMO.

## IV Conclusions

In this work, we have used a generalized Breit-Wigner treatment to compare the transport characteristics of two important types of organic molecules (alkanes and alkenes) as prototype cases of saturated and conjugated structures, respectively. In this formalism, both ballistic and cotunneling processes are allowed to contribute to the overall charge transfer between the electrodes, where cotunneling processes can be associated with changes in the oxidation state of the molecule. We have found that for the alkane molecules considered, ballistic mechanisms dominate at low bias, while counneling processes become more important with the progressive increase in the applied voltage. This becomes evident in the corresponding conductance curves, where at a threshold voltage the cotunneling contributions to the overall transport outweigh the ballistic ones. For the alkene family, on the other hand, the transport is dominated by a ballistic mechanism, with an essentially null contribution of cotunneling mechanisms over the entire operational bias range.

Alkenes exhibit a higher conductance and allow current values larger than alkanes. However, we have found that this occurs only over a specific bias range; therefore, there are operational voltages in which this is not observed. The reason for this most likely resides in the cluster model that we used to model the contact with the electrodes. We have seen that the neutral HOMO and LUMO of the alkenes have a more spatially delocalized distribution than those of the neutral alkanes, favoring a higher conductance. However, the HOMO–LUMO gap is larger for the former than for the latter type of molecules, and as a consequence, in the case of alkanes the frontier MOs can begin to participate in the charge transfer transport at lower bias values as the field intensity is increased. This can be seen as the effect of a first approximation of our formalism that will be refined in future works. Finally, at specific voltage thresholds, the neutral HOMO and LUMO of both alkane and alkene families lie inside the Fermi window, when the greater delocalization character of the alkene HOMO and LUMO responds for a now greater level of both the transverse current and corresponding conductance of these conjugated molecules. An additional effect of increasing voltage is to induce a slight localization of the MOs of the neutral forms of both alkanes and alkanes, resulting in a decrease in the ballistic contributions for the transport; however, for the saturated alkanes, the cotunneling contribution begins to increase at higher voltages, leading to an increment of the corresponding total conductance. It is important to point out that avoided-crossing situations between neighboring MOs of charged alkanes, which we have found to exist at certain voltage values, play an essential role in the predicted transport behavior for the alkane family through no negligible cotunneling processes. In these avoided crossings, there is an increase in the resonance between charged and neutral orbitals, opening a new conduction channel involving these MOs. No avoided crossings were observed for the molecules of the alkene family, and hence, cotunneling processes are not relevant to transport in this type of molecule. The method described here can be extended to analyze changes in the state of charge of other molecular systems with a redox center, such as the phenylenediaminebisthiol molecule.^60^

## Supporting Information Available

Additional details on the current curves, the comparison between the conductance curves of both families, the ballistic and cotunneling transmission functions, and the effect of the avoided crossing in the eigenenergies of charged species (PDF)
