# Supplementary material for: Charge Transport in Conjugated and Saturated Hydrocarbons: Comparing Ballistic and Cotunneling Contributions
Source: J Phys Chem A. 2023 Dec 15;127(51):10828–37. doi: 10.1021/acs.jpca.3c05869 (PMC10911760; doi:10.1021/acs.jpca.3c05869)
Supplement: Supplementary file 1 — jp3c05869_si_001.pdf [file jp3c05869_si_001.pdf]

Charge Transport in Conjugated and Saturated Hydrocarbons: Comparing Ballistic and Cotunneling Contributions

Hugo Cabrera-Tinoco,[1,*] Augusto C. L. Moreira,[2] Luis Borja-Castro,[3] Renato Valencia-Bedregal,[3] Crispin H.W. Barnes,[4] Luis de los Santos Valladares[4,*]

[1] Facultad de Ingeniería, Universidad Continental, Lima 15311, Perú.
[2] Núcleo Interdisciplinar em Ciências Exatas e da Natureza (NICEN), Universidade Federal de Pernambuco, 55014-900 Caruaru – PE, Brazil.
[3]Laboratorio de Cerámicos y Nanomateriales, Facultad de Ciencias Físicas, Universidad Nacional Mayor de San Marcos, Ap. Postal 14-0149, Lima, Perú.
[4]Cavendish Laboratory, Department of Physics, University of Cambridge, J. J Thomson Av., Cambridge CB3 0HE, UK.

*Corresponding authors emails: hcabrera@continental.edu.pe (Hugo Cabrera-Tinoco) and ld301@cam.ac.uk (Luis De Los Santos Valladares)

Supporting Information

SI-I: Comparison AM and GBW

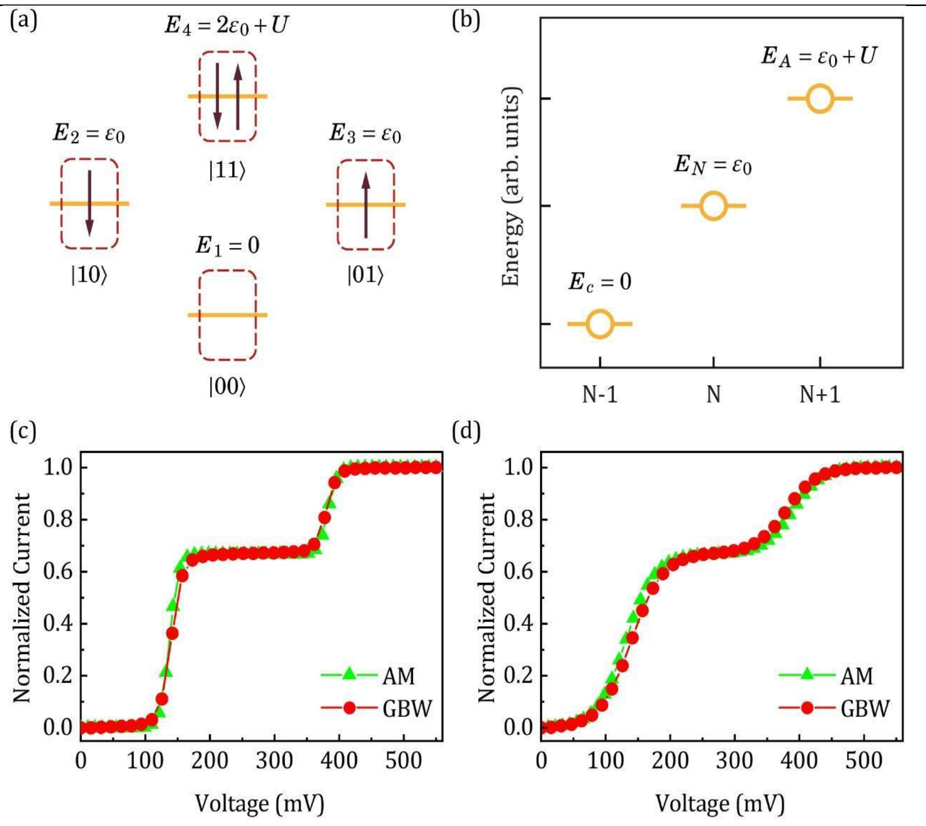

Figure S1: Fock space for the different charge states of an AM impurity (a). GBW structure of a single level system defined to compare with AM (b). I-V curves

generated by the GBW and AM approaches at 40K (c) and 130K (d). The parameters and the set of probabilities ɣ are defined in section 2.

SI-2: Current vs voltage curves

In Fig S2(a) we show the variation of the current calculated by use of Eq. (1) as a function of the applied voltage, for each one of the alkanes examined. In all cases, the current increases considerably at a small bias ($V \leq 0.2V$) and then tends to saturate. As one can observe, the increase in the intensity of the total current is more noticeable in the case of the molecules of smaller size. For example, while the current for butanedithiol (the C 4 alkane) reaches its maximum at 18 nA, in the decanedithiol (C 10) case, saturation occurs at only 2 nA. In Fig. S2(b), we show that at $V > 0.6\ V$ the calculated current for the alkene molecules considered can reach values of the order of 100 to 160 nA, which are almost one order of magnitude higher than those found for the alkane family. However, one can also observe that at lower voltages the current in the alkanes is, in fact, higher than those of the similar alkenes. For example, for $V < 0.47\ V$ the current in the 1,3-butedinedithiol molecule (C 4 alkene) is smaller than that estimated for the C 4 alkane. Similar behavior is found for C 10 alkane relative to the 1,3,5,7,9-desentenedenedithiol molecule (C 10 alkene) for $V < 0.25\ V$.

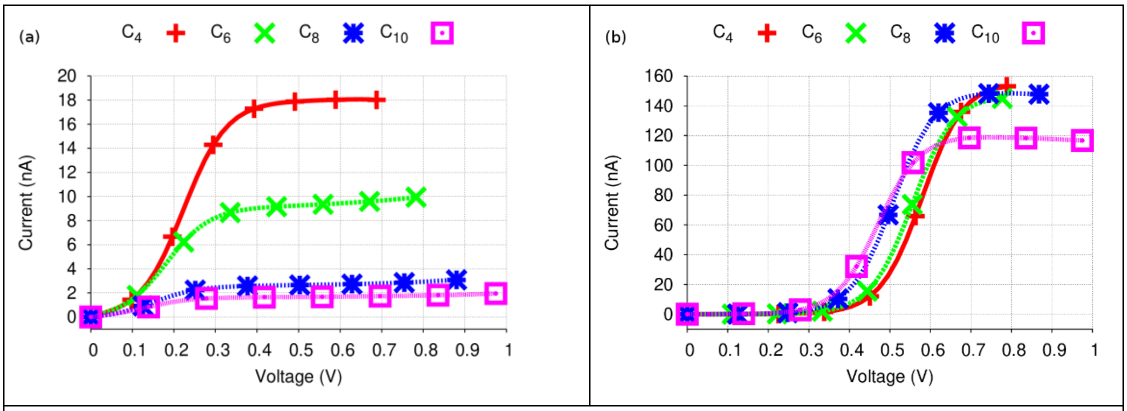

Figure S2: Current versus voltage of the alkane (a) and alkene (b) family.

SI-3: Conductance curves of the alkane and alkene families

In Fig. S3, we present the conductance curves of the C4, C6, C8 and C10 molecules of both hydrocarbon families. As one can observe, although alkenes present a higher conductance at higher voltages, the inverse is true in low fields. As we will discuss later (Fig. 6), this can be attributed to the electronic structure of the corresponding neutral species.

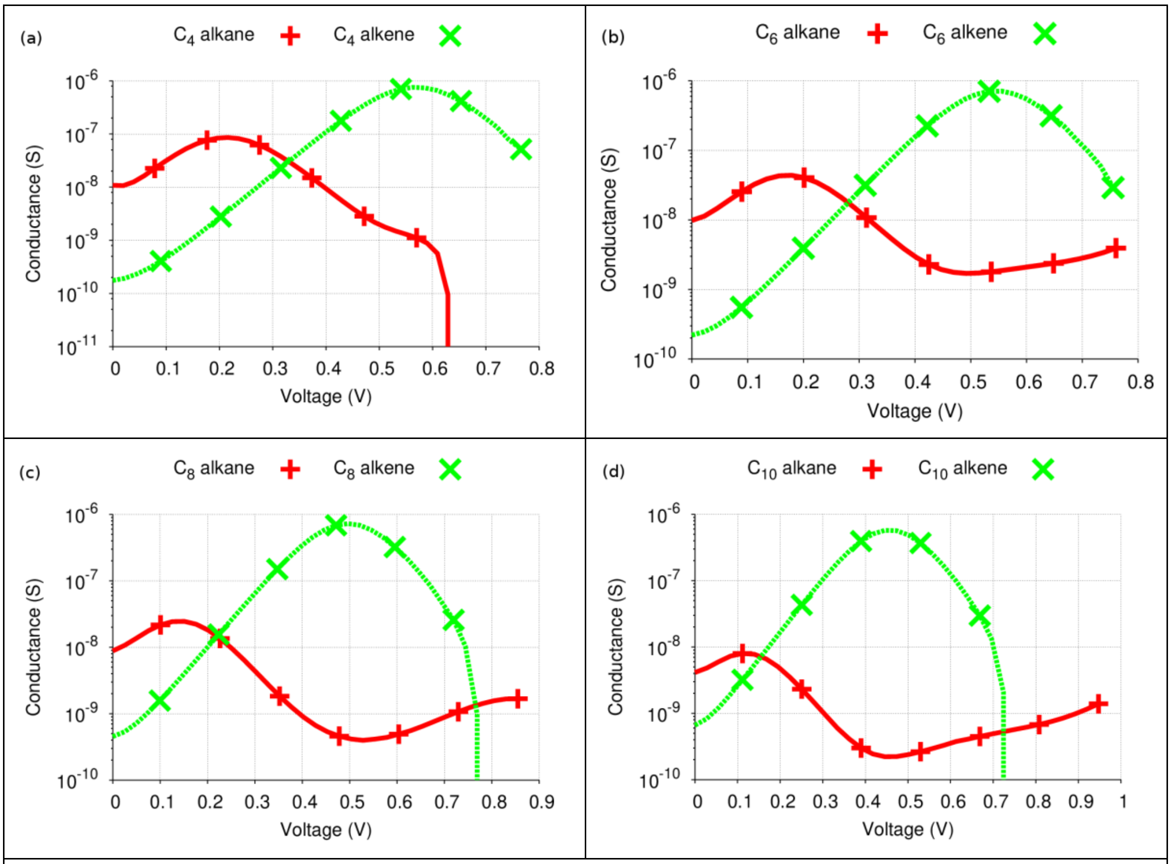

Figure S3: Conductance curves of the C4 (a), C6 (b), C8 (c) and C10 (d) alkanes (red crosses) and alkenes (green exes).

SI-4: Ballistic transmission function

It is possible to identify the molecular orbitals that can participate in the transport as viable charge carrier channels. In Fig. S4(a) we present the energy distribution of the ballistic transmission function of the decanedithiol molecule calculated at 0.97 V. For this voltage, the orbitals of the charged species contribute the most to the cotunneling processes, as we will discuss later. One can observe that the ballistic transmission through the neutral species is six orders of magnitude larger than that of the anionic species. The two peaks in Fig S4(a) correspond to the HOMO and LUMO, as identified by their energy position. Similarly, we can pinpoint which of the molecular orbitals are the more relevant for the ballistic transmission, after we realize the cationic species is not involved in this type of transport since their MOs are entirely localized. We present the corresponding

ballistic current curves in Fig. S4(b), where one can see that the neutral species is indeed the most relevant in the ballistic type of transport.

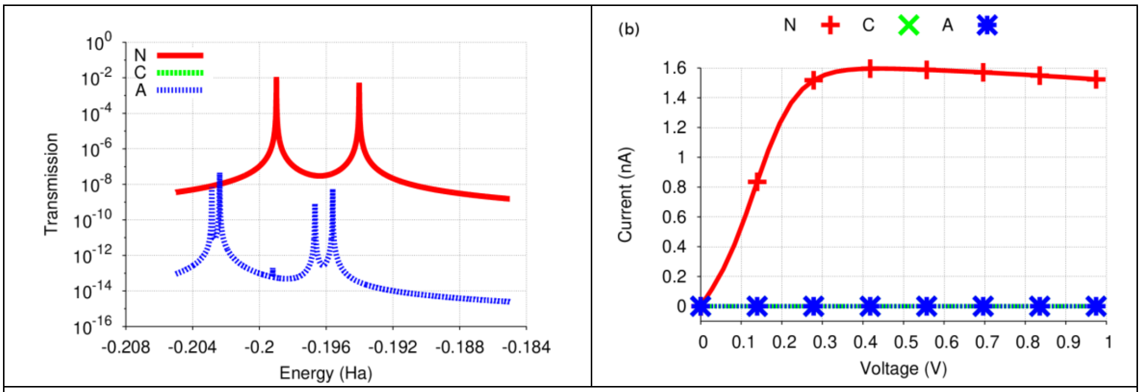

Figure S4: Decanedithiol molecule: a) Ballistic transmission function at 0.97 V. b) Variation of the ballistic current associated with the neutral (N), cationic (C) and anionic (A) species as a function of the applied bias. Since the cation MOs are completely localized, there is no ballistic transmission associated with the cationic species.

SI-5: Cotunneling transmission function

As in the case of ballistic transport, we can identify the molecular orbitals that contribute to transport through the cotunneling mechanism. For this type of process to occur, molecular orbitals of two different states of charge must participate. We can identify which orbitals contribute by verifying their eigenvalues. We will adopt the notation N^AN to describe the cotunneling process in which two MOs of the neutral species behave as transport channels with the assistance of one MO of the anionic species. Inversely, an ANA mechanism corresponds to the case where the orbitals of the anionic species are the ones to behave like channels, assisted by one MO of the neutral species, and so on. In Figs. S5(a) and S5(b), we present the calculated cotunneling transmission function at 0.97 V for the decanedithiol molecule. Examining the peak distribution, one can identify two cotunneling processes: one involving the neutral LUMO (the highest peak in the red continuous curve of Fig. S5(a)) and the anionic $H_A - 13_\alpha$ and $H_A - 12_\beta$ (the highest peaks in the green dashed curve), and another one, in which the neutral HOMO (the highest peak of the blue dashed curve of Fig. S5(b)) participates with the cationic $L_C + 2_\alpha$ and $L_C + 3_\beta$ (the highest peaks in the purple continuous curve).

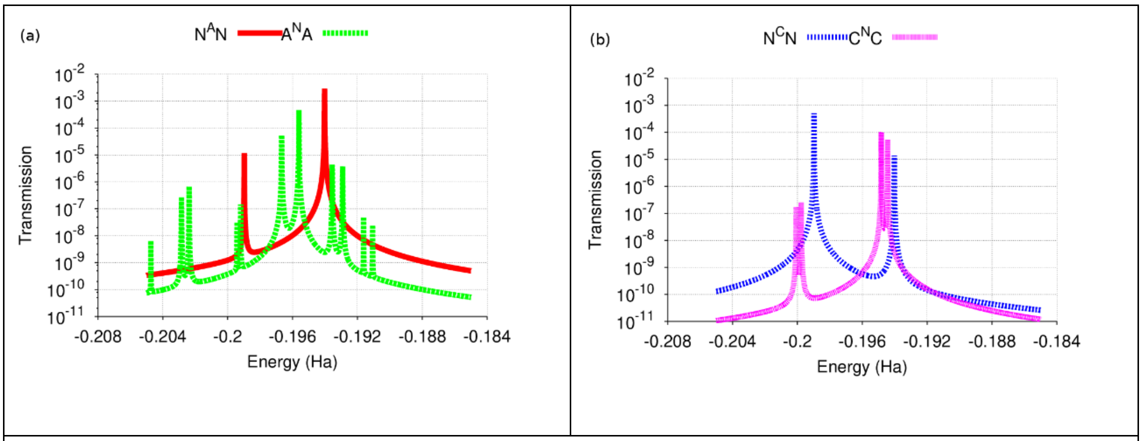

Figure S5: Energy distribution of the cotunneling transmission function of the decaneditiol molecule corresponding to the joint participation of the anionic and neutral species (a) and the cationic and neutral species (b).

SI-6: Alkane and alkene molecular orbitals differences

As discussed, cotunneling mechanisms are not relevant for the charge transport of the alkene molecules due to the low degree of resonance between the MOs of the neutral and charged species in this family. As the voltage is switched on, avoided-crossing situations develop for some of the MOs of both the cationic and anionic species, resulting in an increase in the degree of resonance of these orbitals with the MOs of the neutral species. However, no avoided-crossings exist for the MOs in any of the three charged species of the alkene molecules. In Fig. S6, we show the eigenvalues of the MOs of the anionic and neutral species of the decanedithiol (C10 alkane) and 1,3,5,7,9-decepentenedithiol (C10 alkene) molecules. The $H_A - 9_\alpha$ (Fig. S6(a)) and $H_A - 9_\beta$ (Fig. S6 (b)) of the C10 alkene have an adequate spatial localization but a low degree of resonance with the neutral LUMO (see discussion of Fig. 5), and this situation remains unchanged when the voltage is turned on. Hence, cotunneling processes will not be favored.

On the other hand, as the voltage is increased, the energies of the $H_A - 15_\alpha$, $H_A - 14_\alpha$ and $H_A - 13_\alpha$ alpha MOs and $H_A - 13_\beta$, $H_A - 12_\beta$ and $H_A - 11_\beta$ beta MOs of the alkane C10 evolve in such manner as to sequentially approach the energy of the neutral LUMO. As a result, an effective conducting channel involving the MOs of the anion form

(Ch_A) is established (purple squares in Fig. S6), due to the sequence of avoided-crossing events (see discussion of Fig. 7).

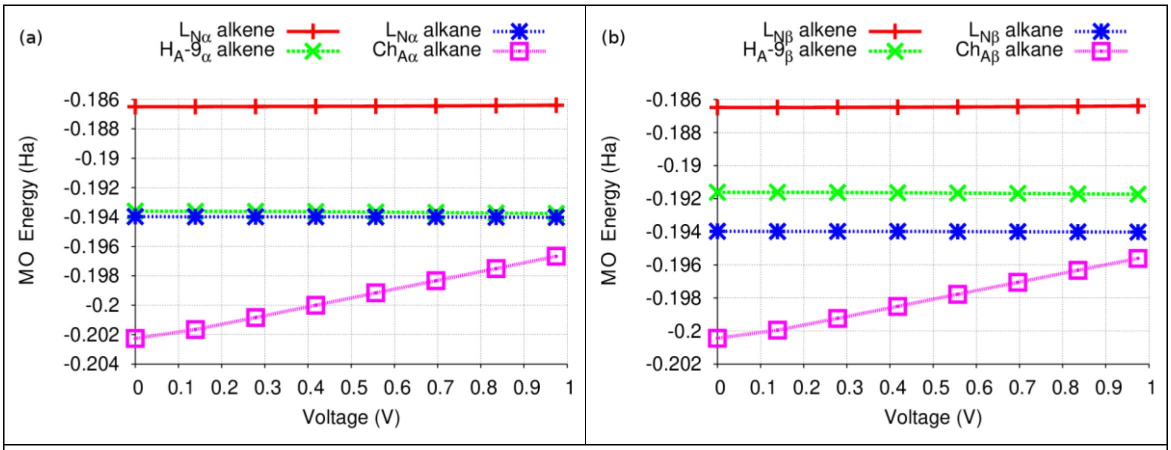

Figure S6: Evolution of the energy of alpha (a) and beta (b) orbitals for the C10 alkane and C10 alkene molecules with increasing voltages: neutral LUMO (L_N), anionic HOMO-9 ($H_A - 9$) and the alkane effective conducting channel (Ch_A).
